# SLAP repair with arthroscopic decompression of spinoglenoid cyst

**DOI:** 10.1051/sicotj/2015036

**Published:** 2016-01-22

**Authors:** Hiroshi Hashiguchi, Satoshi Iwashita, Atsushi Ohkubo, Shinro Takai

**Affiliations:** 1 Department of Orthopaedic Surgery, Nippon Medical School Chiba Hokusoh Hospital 1715 Kamakari Inzai, Chiba 270-1694 Japan; 2 Department of Orthopaedic Surgery, Nippon Medical School Hospital 1-1-5 Sendagi Bunkyo-ku, Tokyo 113-8602 Japan

**Keywords:** Spinoglenoid cyst, Suprascapular nerve, Arthroscopic decompression, Labral tear, SLAP repair

## Abstract

*Introduction*: A spinoglenoid cyst with suprascapular nerve disorders is highly associated with superior labrum anterior posterior (SLAP) lesion. Conservative or surgical treatment is applied to relieve pain and neurological symptoms. The purpose of this study was to evaluate clinical outcomes of patients treated by arthroscopic surgery for SLAP lesion with a spinoglenoid cyst.

*Methods*: The subjects of this study were six patients with SLAP lesion with a spinoglenoid cyst who underwent arthroscopic surgery. There was one female and five males with a mean age of 48.5 years. SLAP lesion was found in all the patients at arthroscopy. A small tear of the rotator cuff was found in the two patients. The SLAP lesion was repaired using suture anchors, and the rotator cuff tears were repaired by suture-bridge fixation. The spinoglenoid cyst was decompressed through the torn labrum in three patients, and through the released superior to posterior portion of the capsule in the other three patients.

*Results*: All patients showed excellent improvement in pain and muscle strength at the final follow-up examination. The mean Constant score was improved from 60.5 points preoperatively to 97.2 points postoperatively. The mean visual analog scale (VAS) score decreased from 4.5 on the day of the surgery to 2.5 within one week postoperatively. Postoperative MRI showed disappearance or reduction of the spinoglenoid cyst in four and two patients, respectively. There were no complications from the surgical intervention and in the postoperative period.

*Discussion*: The patients treated by decompression through the released capsule obtained pain relief at an early period after the surgery. Arthroscopic treatment for a spinoglenoid cyst can provide a satisfactory clinical outcome. Arthroscopic decompression of a spinoglenoid cyst through the released capsule is recommended for a safe and reliable procedure for patients with suprascapular nerve disorders.

## Introduction

The suprascapular nerve is a branch of the upper trunk of the brachial plexus which is derived from spinal nerves C4-6. The nerve passes along the medial aspect of the base of the scapular coracoid process and then between the transverse scapular ligament and the suprascapular notch to innervate the supraspinatus and infraspinatus muscles. Compression of the suprascapular nerve leads to pain, limitation in external rotation of the shoulder, muscle weakness, supraspinatus and infraspinatus muscle atrophy, and other neurological symptoms [1, 2]. Suprascapular neuropathy is typically caused by thickening of the transverse scapular ligament or a spinoglenoid cyst [3, 4]. A spinoglenoid cyst is a ganglion arising in the spinoglenoid notch [5, 6]. Because it is often accompanied by labral tears such as superior labrum anterior posterior (SLAP) lesion, it is considered to be formed by the one-way valve mechanism where synovial fluid is leaked through the torn labrum and accumulates to form a cyst [7, 8]. A spinoglenoid cyst can be treated either conservatively or surgically. Conservative treatment consists of symptomatic therapy to relieve neurological symptoms, such as medication, and follow-up observation. While spontaneous resolution or reduction of a cyst with subsequent improvement in symptoms has been achieved in some cases, there have also been patients who have not responded to conservative treatments due to recurrent cysts or worsening of symptoms due to an enlarging cyst [9]. Surgical treatment may be selected for early improvement of symptoms or prevention of recurrence and can be performed either by open surgery or arthroscopically [10–13]. Open surgery enables reliable decompression by removal of a spinoglenoid cyst, but is highly invasive due to dissection of the deltoid muscle. Arthroscopic surgery has been increasingly selected as a minimally invasive and reliable technique. Arthroscopic procedures are divided into decompression of the cyst, and repair of the torn labrum that forms a one-way valve.

The purpose of our study was to report our outcomes of patients treated by arthroscopic surgery for SLAP lesion with a spinoglenoid cyst.

## Materials and methods

This study was approved by the Institutional Review Board of our hospital. A single surgeon performed arthroscopic surgery on six patients with suprascapular neuropathy associated with a spinoglenoid cyst between 2007 and 2012. Those patients who were not followed up for at least six months after surgery or did not undergo postoperative magnetic resonance imaging (MRI) were excluded from the analysis. Patients who had taken part in overhead sports such as baseball were not included in this series.

**Figure 1.**
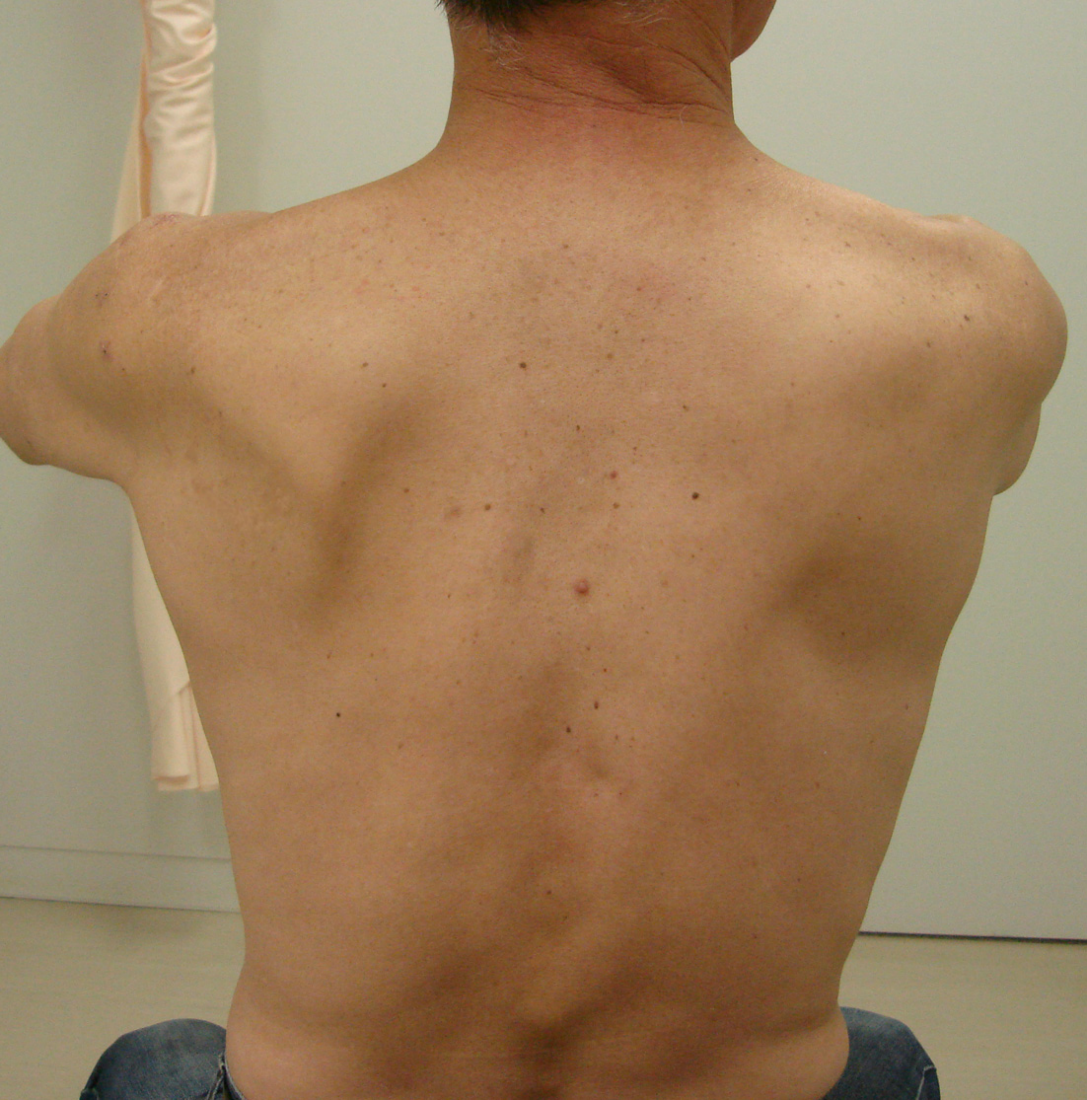
Atrophy of the left infraspinatus muscle caused by suprascapular neuropathy with a spinoglenoid cyst.

**Figure 2.**
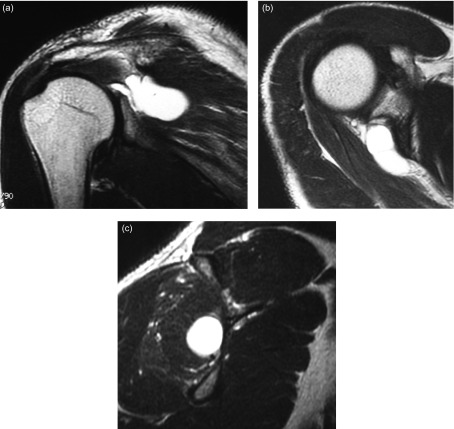
T2-weighted MRI scans of a left shoulder demonstrating a spinoglenoid cyst on the coronal image (a), the axial image (b), and the sagittal image (c). The sagittal image shows edema and atrophy of the infraspinatus muscle.

Preoperative complaints included pain or dullness in the posterior or deep shoulder in all patients, as well as pain at rest and night pain in some patients. Physiological findings included infraspinatus muscle atrophy (Figure 1) and tenderness of the muscle belly, limited shoulder range of motion and muscle weakness, which were observed in all patients. No abnormality was evident on plain X-ray. MRI revealed a cyst that showed low intensity on T1-weighted images and high intensity on T2-weighted images (Figure 2), extending from the postero-superior labrum to the spinoglenoid notch, as well as a small tear of the supraspinatus tendon in two patients. T2-weighted sagittal images showed edema and atrophy of the infraspinatus muscle in all of the patients. SLAP lesion confirmed connection to a cyst which was detected in five patients. Electromyography (EMG) was performed in two patients. EMG findings of the two patients showed denervation pattern of the infraspinatus muscle only. One patient underwent needle aspiration of a cyst under ultrasound guidance after the definitive diagnosis, but there was no improvement in symptoms. For the remaining five patients, surgical treatment was selected after the definitive diagnosis.

### Operative techniques

**Figure 3.**
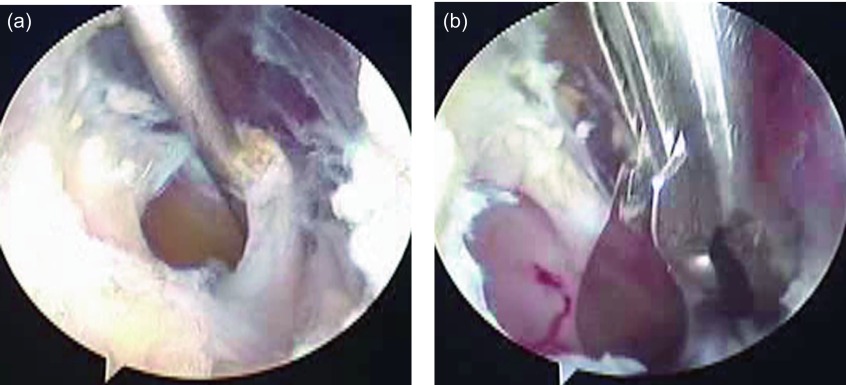
Arthroscopic imaging demonstrating decompression of the spinoglenoid cyst through the released superoposterior capsule (a), and partial removal of the cyst using an arthroscopic punch (b).

Patients were positioned in a lateral decubitus position using a traction sling under general anesthesia. Diagnostic arthroscopy was performed through a posterior portal into the glenohumeral joint to examine SLAP lesion and other intra-articular pathology. SLAP lesions were confirmed as type II in all of the patients. In three patients, SLAP lesion was expanded in the anteroposterior direction to perform extravasation of jelly-like fluid in the cyst. In the other three patients, removal of the cyst was performed by making a release of the posterior to superior capsule over the cyst to identify the cyst, followed by evacuation of the cyst septations to drain the fluid (Figure 3). The released capsule was left unrepaired. In all the patients, the SLAP lesion was treated by footprint debridement and decortication, and repaired with one or two PANALOK absorbable suture anchors (Depuy-Mitek, Warsaw, IN). Two patients with a small tear of the supraspinatus tendon were treated with suture-bridge repair technique using a Corkscrew FT metal suture anchor (Arthrex, Naples, FL) and two VERSALOK suture anchors (Depuy-Mitek). Release of the spinoglenoid ligament was not performed in all of the patients.

After the surgery, a sling was used to immobilize the shoulder for two weeks. Physical therapy was started on the second postoperative day. The program started with pendulum exercise and passive range of motion exercises of the shoulder. The sling was removed two weeks postoperatively and then active exercise was started. The two patients who underwent rotator cuff repair started muscular strength training six weeks postoperatively, while the other four patients started functional rotator cuff training two weeks postoperatively. Patients were allowed to return to heavy work or sports activities in approximately two months after the surgery, after improving shoulder motion and muscle strength.

### Clinical and imaging evaluation

Patients were subjected to regular clinical follow-up starting from the preoperative period through to the final follow-up examination. In all patients, clinical outcome was evaluated using the Constant shoulder score, while pain assessment from the day of surgery to one week after the operation was performed using the visual analog scale (VAS). All patients underwent MRI three months (2.5–4 months) postoperatively, after patients obtained full range of motion and normal muscle strength of their affected shoulder.

## Results

We treated one female and five males, with a mean age of 48.5 (34–70) years (Table 1). Four of the six patients became symptomatic without a known cause, while one patient had a history of fall and another patient had lifted a heavy object before becoming symptomatic. Of the four patients who became symptomatic without a known cause, one had been diagnosed with cervical spondylotic radiculopathy and had received treatment. The mean symptomatic period was 3.1 months (range, 0.5–6 months).

**Table 1.** Patient data.

Patient no.	Age (years)	Sex	Cyst size (cm)	Decompression procedure	Constant score		VAS		Follow-up (m)
					Preop	Postop	Preop	Postop	
1	39	Male	4.3 × 2.2	TL	51	100	6	1	42
2	70	Female	2.0 × 1.0	TL	62	94	8	3	34
3	59	Male	4.0 × 1.8	RC	59	98	7	0	67
4	34	Male	1.2 × 1.0	TL	63	100	10	4	64
5	44	Male	3.4 × 2.0	RC	68	98	8	1	92
6	45	Male	2.8 × 1.6	RC	60	96	7	0	85

**Figure 4.**
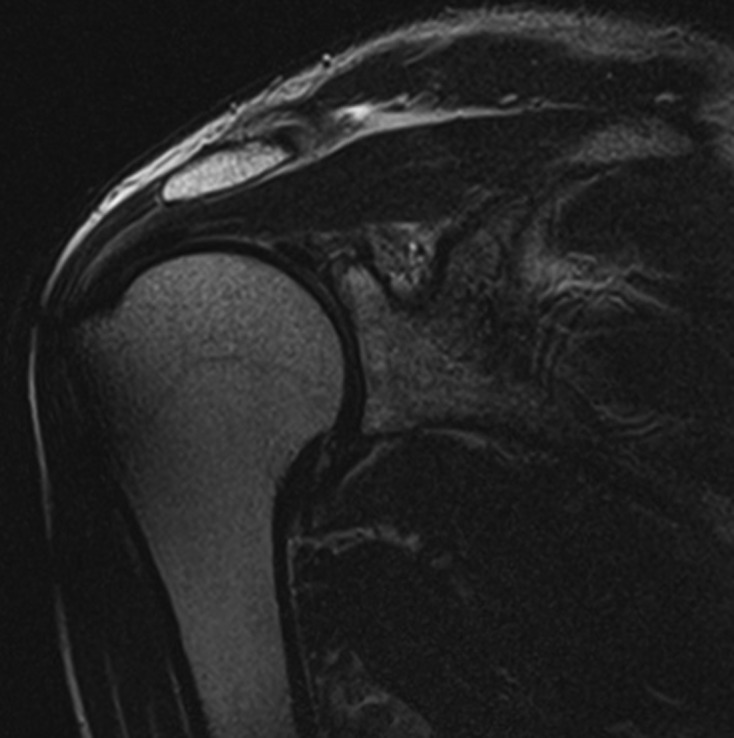
Postoperative T2-weighted MRI scan demonstrating disappearance of the spinoglenoid cyst.

All patients underwent preoperative MRI for localization of the spinoglenoid cyst. The cyst size ranged from 1 to 4 cm. Edema and atrophy of the infraspinatus muscle belly was observed in three patients (Figure 2). Postoperative MRI showed the disappearance or reduction of the spinoglenoid cyst in four and two patients, respectively. Disappearance of the cyst was achieved in all the three patients treated by decompression of the cyst through the released capsule (Figure 4). Although edema of the infraspinatus muscle was improved in all of the patients, muscle atrophy remained in four patients whose symptomatic period was more than three months.

**Figure 5.**
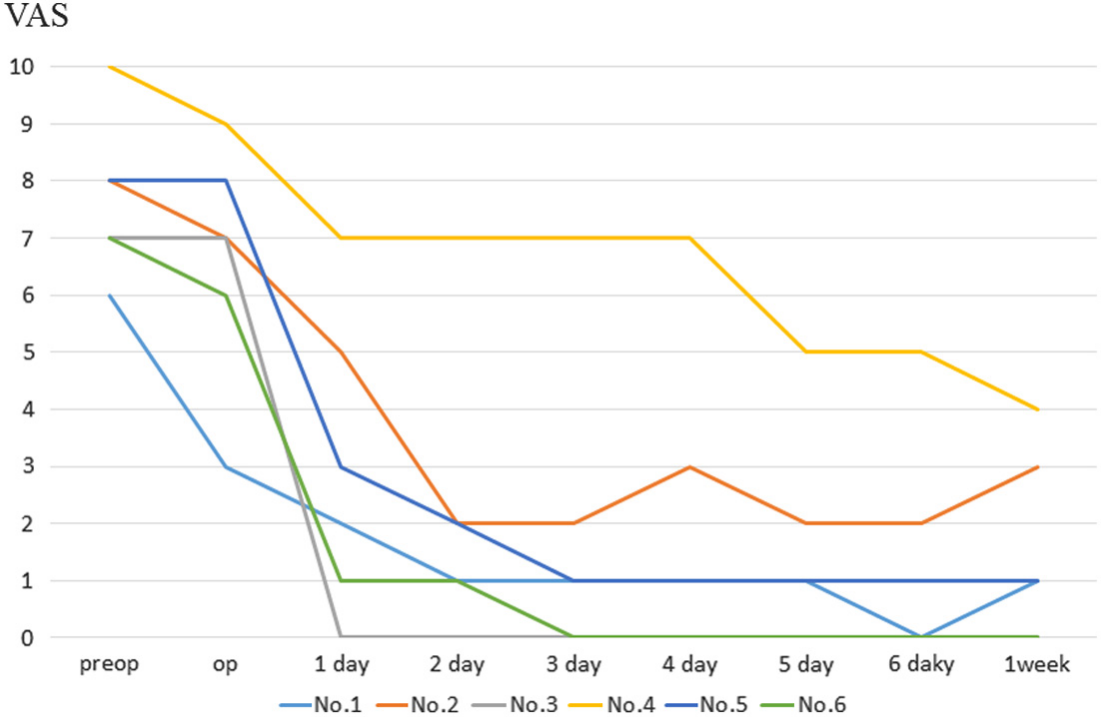
The VAS score of the three patient (no. 3, 5, 6) treated by decompression of the cyst through the released capsule decreased earlier than those through the torn labrum.

The mean postoperative follow-up period was 63.7 months (range, 34–92 months). The mean Constant score was improved from 60.5 (range, 51–68) preoperatively to 97.2 (range, 96–100) postoperatively. The mean time to pain resolution after the surgery was 7.2 weeks (range, 1 day to 20 weeks). All patients had normalized muscle strength at the final follow-up examination. The mean VAS score decreased from 7.7 (range, 6–10) preoperatively to 1.5 (range, 0–5) one week postoperatively (Figure 5). The mean VAS score for the three patients treated by decompression of the cyst through the released capsule decreased to 1.0 the day after the surgery, suggesting that earlier pain relief was achieved in these patients compared with those treated by decompression through the torn labrum.

No complications, such as nerve injury, hematoma, and infection, were observed during follow-up. No patient required revision surgery.

## Discussion

Previous articles have reported that a spinoglenoid cyst is frequently associated with superior labral lesion, even if not evident on MRI. Tirman et al. [8] stated that an abnormality of the glenoid labrum associated with a cyst is observed in all patients. Kim et al. [14] stated that a preoperative MRI revealed SLAP lesion associated with spinoglenoid cyst in 24 of 28 patients. Piatt et al. [15] reported that the incidence of ganglion cyst and SLAP lesion is 89%. In this study, preoperative MRI revealed evidence of labral pathology in five of the six patients, although a type II SLAP lesion was observed by diagnostic arthroscopy in all of the patients.

EMG evaluation is helpful to diagnose suprascapular neuropathy in patients with a spinoglenoid cyst. EMG abnormalities of the supraspinatus and infraspinatus muscles suggest suprascapular nerve compression proximally at the suprascapular notch. The presence of involvment of the infraspinatus muscle only suggests compression at the spinoglenoid notch. EMG evaluation of the two patients in this study revealed denervation pattern of the infraspinatus muscle only. However, the EMG may show false-negative findings even in patients with suprascapular neuropathy. Innervation of the nerve branches to the infraspinatus muscle occurs in false-negative findings and multiple location of EMG evaluation is necessary to localize the lesion [7]. Therefore, definitive diagnosis of suprascapular neuropathy should be obtained by symptoms, physical findings, EMG evaluation, and MRI findings such as edema and atrophy of the muscles.

A spinoglenoid cyst can be treated either conservatively or surgically. Conservative treatment includes follow-up observation with symptomatic treatment by medication, and needle aspiration under ultrasound or computed tomography (CT) guidance [9]. Although spontaneous disappearance can be achieved by conservative treatment in some patients, some patients experience worsening of paralysis due to an enlarging cyst and thus require close follow-up. Needle aspiration under CT or ultrasound guidance has been associated with favorable outcomes, but it has also been associated with a poor outcome due to insufficient decompression and recurrence [15]; therefore, the reliability of the technique is poor.

Among surgical treatments, open removal of the cyst enables reliable cyst removal, but it is highly invasive due to the need for an extensive skin incision and dissection of the deltoid muscle and is also associated with the risk of postoperative contracture and weakness of the deltoid muscle [13].

Since it was first reported by Iannotti and Ramsey [16], arthroscopic surgery has been increasingly selected as a minimally invasive and reliable technique. Arthroscopic procedures include cyst decompression and repair of a labral tear. Labral repair alone has been associated with poor outcomes and poor patient satisfaction [14, 17–19]. Poor outcome after labral repair alone is likely to be due to the extended time required for achieving cyst reduction, resulting in persistent nerve compression and associated pain. This implies the importance of cyst decompression. Cyst decompression can be achieved either through the torn labrum or through the released capsule [20–23]. Decompression through the torn labrum allows for drainage of fluid without damaging surrounding tissue, but it is associated with the risk of incomplete drainage. The three patients treated by this approach also experienced persistent pain after the surgery, with postoperative MRI showing reduced but residual cysts in two of the three patients. In contrast, decompression of the cyst through the released capsule enables complete drainage of the cyst fluid and immediate nerve decompression. The three patients treated by decompression of the cyst through the released capsule obtained nearly complete resolution of pain the day after the surgery. Postoperative MRI also confirmed disappearance of spinoglenoid cyst in all these patients. Arthroscopic decompression of the cyst can be done by either the intra-articular or subacromial approach [24, 25]. Decompression by the subacromial approach is applicable not only to spinoglenoid cysts, but also to suprascapular cysts, and is thus considered a safe and reliable decompression technique. However, additional portals are required to treat intra-articular lesions, such as the torn labrum. This approach is also technically challenging and requires a learning curve. Decompression by the intra-articular approach is not applicable to suprascapular cysts and requires capsular release. An advantage of this approach is that both removal of a spinoglenoid cyst and repair of the torn labrum can be performed through the same portals. This technique is also associated with a lower risk of nerve injury and technically less challenging so that it can be performed by standard shoulder arthroscopy skills. This technique eliminates the risk of complications from capsular release and is also effective in preventing recurrence of a spinoglenoid cyst with combination of repair of the labral tear.

In conclusion, a SLAP lesion with a spinoglenoid cyst is rare, but should be recognized as a condition that induces pain and impaired function of the shoulder. A patient with suprascapular nerve disorder associated muscle atrophy and weakness should undergo MRI as soon as possible to obtain a definitive diagnosis. Surgical treatment should be considered if conservative treatment is not effective. Decompression by the intra-articular approach is a safe and effective surgical procedure that is likely to lead to satisfactory outcomes.

## Conflict of interest

The authors declare that they have no conflict of interest.
